# Sequence, "subtle" alternative splicing and expression of the *CYYR1 *(*cysteine/tyrosine-rich 1*) mRNA in human neuroendocrine tumors

**DOI:** 10.1186/1471-2407-7-66

**Published:** 2007-04-18

**Authors:** Lorenza Vitale, Flavia Frabetti, Shane A Huntsman, Silvia Canaider, Raffaella Casadei, Luca Lenzi, Federica Facchin, Paolo Carinci, Maria Zannotti, Domenico Coppola, Pierluigi Strippoli

**Affiliations:** 1 Department of Histology, Embryology and Applied Biology, University of Bologna, Bologna, Italy; 2 "H. Lee Moffitt" Cancer Center, University of South Florida, Tampa, Florida, USA

## Abstract

**Background:**

*CYYR1 *is a recently identified gene located on human chromosome 21 whose product has no similarity to any known protein and is of unknown function. Analysis of expressed sequence tags (ESTs) have revealed high human *CYYR1 *expression in cells belonging to the diffuse neuroendocrine system (DNES). These cells may be the origin of neuroendocrine (NE) tumors. The aim of this study was to conduct an initial analysis of sequence, splicing and expression of the *CYYR1 *mRNA in human NE tumors.

**Methods:**

The *CYYR1 *mRNA coding sequence (CDS) was studied in 32 NE tumors by RT-PCR and sequence analysis. A subtle alternative splicing was identified generating two isoforms of *CYYR1 *mRNA differing in terms of the absence (CAG^- ^isoform, the first described mRNA for *CYYR1 *locus) or the presence (CAG^+ ^isoform) of a CAG codon. When present, this specific codon determines the presence of an alanine residue, at the exon 3/exon 4 junction of the *CYYR1 *mRNA. The two mRNA isoform amounts were determined by quantitative relative RT-PCR in 29 NE tumors, 2 non-neuroendocrine tumors and 10 normal tissues. A bioinformatic analysis was performed to search for the existence of the two *CYYR1 *isoforms in other species.

**Results:**

The *CYYR1 *CDS did not show differences compared to the reference sequence in any of the samples, with the exception of an NE tumor arising in the neck region. Sequence analysis of this tumor identified a change in the CDS 333 position (T instead of C), leading to the amino acid mutation P111S. NE tumor samples showed no significant difference in either *CYYR1 *CAG^- ^or CAG^+ ^isoform expression compared to control tissues. *CYYR1 *CAG^- ^isoform was significantly more expressed than CAG^+ ^isoform in NE tumors as well as in control samples investigated. Bioinformatic analysis revealed that only the genomic sequence of *Pan troglodytes CYYR1 *is consistent with the possible existence of the two described mRNA isoforms.

**Conclusion:**

A new "subtle" splicing isoform (CAG^+^) of *CYYR1 *mRNA, the sequence and the expression of this gene were defined in a large series of NE tumors.

## Background

The cysteine/tyrosine-rich 1 gene (*CYYR1*) is a large gene of 107 kb that we have previously identified [1] on human chromosome 21 (21q21.2). *CYYR1 *is composed of 4 small exons separated by three large introns and it encodes a small 154-amino acid polypeptide conserved in vertebrates. The most prominent feature of the protein family is the presence of a central cysteine- and tyrosine-rich domain, highly conserved from fish to humans and including a CCSYYAY box. There is also a proline-rich region, localized at carboxy-terminus, consisting of three repeated PPPY motifs. A putative transmembrane domain was identified in all proteins of the family by different prediction methods, along with a signal peptide suggesting the possible location of the CYYR1 protein in the cell or cell compartment membrane. *CYYR1 *product has no similarity to any known protein and its function is unknown.

Two different *CYYR1 *mRNA species were identified using Northern blot analysis: they were consistent with two isoforms arising from alternative polyadenylation. The expression pattern of the *CYYR1 *human gene appears to be broad, as *CYYR1 *mRNA was detected in all 12 human tissues tested, except peripheral blood leukocytes [1].

Valuable data about the function of the *CYYR1 *gene were added by human expressed sequence tag (EST) database analysis. This was possible because the main source of tissues and cells used in the construction of libraries, from which most of the *CYYR1*-related ESTs were retrieved, included carcinoid tumor of the lung, melanocytes, parathyroid tumor cells, fetal adrenal tissue, follicular carcinoma of the thyroid, pineal gland and gastrointestinal tissue. A common feature of all these tissues is that they are composed of or include cell types belonging to the dispersed neuroendocrine system (DNES). The term "neuroendocrine" has been used to define cells such as neurons and endocrine cells that secrete their products in a regulated manner in response to a specific stimulus, and share a common phenotypic program, characterized by the expression of specific markers such as neuropeptides and chromogranins [2]. These cell types are typical of vertebrates and the EST expression data are consistent with the *CYYR1 *phylogenetic analysis.

It is assumed that NE tumors can originate from normal NE cells. NE tumors are divided, according to the WHO (World Health Organization) classification, into well-differentiated tumors or carcinomas, poorly differentiated carcinomas and mixed endocrine-exocrine carcinomas [3], according to their histological and cytological features.

The aim of this study was to analyze the sequence, splicing and expression of the *CYYR1 *gene in human neuroendocrine tumors. First, the *CYYR1 *mRNA coding sequence (CDS) was studied in a large series of NE tumors and a P111S mutation in the only neck-derived tumor was identified. Sequence analysis also allowed us to identify a "subtle" splicing isoform due to the existence of two functional acceptor splicing sites in the sequence CAGCAG at the 3' end of intron 3. Both isoforms, which encode two polypeptides – differing in terms of the absence or presence of one amino acid – were found in normal and neoplastic tissues. Their relative expression was investigated by a quantitative relative RT-PCR (reverse transcription – polymerase chain reaction) method.

## Methods

### Sample collection

Sample selection was focused on neuroendocrine tumors from multiple anatomic sites (mostly from the digestive system) with varying differentiation grades (see Additional file 1). Tumors were classified according to the WHO classification of endocrine tumors [3]. The specimens were obtained from 16 males and 11 females ranging between 43 and 79 years of age (mean age of 63.6 years). We used representative portions of 32 tumor specimens (see Additional file 1) labelled NE 1 to NE 32 (15 samples: well differentiated endocrine carcinomas, WDEC; 13 samples: poorly differentiated endocrine carcinomas, PDEC; 4 samples: mixed exocrine-endocrine tumors, MEET). Samples were collected for the H. Lee Moffitt Cancer Center and Research Institute (Tampa, FL) Tissue Procurement Facility, under institutional review board protocols. The resection-to-preservation (freezing) time was kept to less than twenty minutes. Sample storage consisted of liquid nitrogen (n = 30) and -80°C (n = 2) for a mean storage period of 41.65 months. Prior to RNA isolation, twelve of these tumor samples underwent independent pathological review by a single pathologist (DC) and were macrodissected while frozen to select tumor-rich areas and decrease the amount of stroma and non-neoplastic elements surrounding the target tumor tissue.

### RNA sources

Total RNA from the NE samples was extracted using the TRIzol (Invitrogen Corp., Carlsbad, CA) according to the manufacturer's protocol. Due to the absence of tissues entirely composed of DNES cells, which are by definition part of a diffuse system of cells, a set of 8 commercial total RNA samples from several whole normal human organs was used as control: prostate (pool of 16 normal adult whole prostates), brain (one normal adult whole brain), heart (one normal adult whole heart), colon (pool of 2 normal adult colons), small intestine (pool of 2 normal adult small intestines), stomach (pool of 15 normal adult stomachs), thymus (pool of 13 normal adult thymuses) and skeletal muscle (pool of 2 normal adult skeletal muscles). All RNA samples were purchased from BD Biosciences Clontech (Palo Alto, CA, USA).

In order to compare the RNA expression of some NE tumor samples (metastatic liver, pancreas and breast tumors) with non-neuroendocrine tumors and corresponding normal tissues, commercial total RNAs from 2 non-neuroendocrine tumors (pancreas and breast tumors) and from the 2 corresponding normal tissues were used in a second experiment. The non- neuroendocrine tumor total RNAs were obtained from a pancreas acinar cell carcinoma (T2N0M0, stage 1B) and from a breast invasive ductal carcinoma (T4N1M0, stage 3B), and were purchased from Ambion (Austin, TX, USA). The 2 normal total RNAs were from a pancreas (normal human pancreas pooled from a 35-year-old Caucasian male) and a breast (normal adult mammary gland pooled from a 27-year-old Caucasian female), and were purchased from BD Biosciences Clontech.

RNA was quantified using both UV spectrophotometry and standard agarose gel. Quantification of electrophoresed RNA was obtained by comparison with standard markers using the gel imaging system Gel Doc 2000 (Bio-Rad, Hercules, CA, USA).

### Primer design

The primers for amplifications were designed using the Amplify software [4], following standard criteria [5]. The data source for primer design was the GenBank sequence AP001696, *Homo sapiens *genomic DNA, chromosome 21 q, section 40/105 [GenBank:AP001696] and NM_052954, *Homo sapiens *cysteine/tyrosine-rich 1 (*CYYR1*) mRNA [GenBank:NM_052954].

For the CDS sequence analysis, we designed the forward primer #1 5'-GCTGCTCTCTCCATCTGATCGC-3' (based on exon 1) and the reverse primer #2 5'-ATTCCAGGCAAGATCGCCCATTG-3' (based on exon 4). The size of the expected PCR product was 605 bp.

For the quantitative relative *CYYR1 *CAG^-^/CAG^+ ^analysis, we designed three primers: a forward primer common to both mRNA forms (#3 5'-GTCTTGCTTCCGAAGTTGGTCCTGC-3'), based on exon 1, and two specific reverse primers for CAG^- ^and CAG^+ ^forms, respectively. Each reverse oligonucleotide was based on the exon 3/exon 4 boundary, and was specific for only one of the two isoforms, containing two mismatches at the last three bases of the 3' end, one being the 3'-residue compared to the sequence of the other isoform (#4 5'-GTGACCGTAGGGTGGTGGTCCAGG-3' for *CYYR1 *CAG^- ^form, primer #5 5'-GTGACCGTAGGGTGGTGGTCCTGC-3' for *CYYR1 *CAG^+ ^form). The expected size of PCR product was 321 bp with primers #3 and #4, and 324 bp with primers #3 and #5.

To amplify the beta-2 microglobulin (*B2M*) housekeeping gene for RNA quantity normalization in quantitative relative RT-PCR analysis [6], we used forward primer #6 5'-GCGGGCATTCCTGAAGCTGACAGCA-3' and reverse primer #7 5'-TACATCAAACATGGAGACAGCACTC-3', with an expected PCR product size of 586 bp.

### RT-PCR

For all samples, total RNA (2 μg) was reverse transcribed at 37°C for 60 min in 50 μL of final volume using cloned Moloney murine leukemia virus reverse-transcriptase 400 U (Promega, Madison, WI; used with companion buffer), 2.5 μM oligo dT-15, 2 μM random hexamers and 500 μM of each dNTP (deoxyribonucleotide triphosphate).

PCR experiments to obtain amplicons for sequence analysis were performed in 50 μL final volume, containing 5 μL reverse transcription mix, 1 U Taq Polymerase (TaKaRa, Shiga, Japan) with companion reagents (0.2 mM of each dNTP, 2 mM MgCl_2_, 1× PCR buffer), and 0.3 μM of each primer. An initial denaturation step of 2 min at 94°C was followed by amplification for 40 cycles, (30 sec at 94°C, 30 sec at 63°C, 45 sec at 72°C) and final extension for 7 min at 72°C.

PCR experiments for quantitative relative analysis were performed in 30 μL of final volume, containing 3 μL of reverse transcription mix, 0.6 U of Taq Polymerase (TaKaRa, Shiga, Japan) with companion reagents (0.2 mM of each dNTP, 2 mM MgCl_2_, 1× PCR buffer), and 0.2 μM of each primer.

To standardize all PCR reactions for quantitative relative analysis, we prepared a single mix with PCR buffer 1×, dNTPs, MgCl_2 _and Taq Polymerase. Subsequently, this mix was dispensed into three aliquots and a different primer pair was added to each one. The cDNA samples were added after each mix has been divided into individual tubes. Each PCR reaction was performed in duplicate. In preliminary PCR experiments, we evaluated PCR products after 20, 25, 30, 35, 40 and 45 cycles (data not shown), in order to find the conditions allowing the quantification of *B2M*, *CYYR1 *CAG^- ^and *CYYR1 *CAG^+ ^RT-PCR products, respectively, at the maximum distance from the cycle corresponding to the reaction plateau. PCR reactions were performed with high stringency: an initial denaturation step of 2 min at 94°C was followed by amplification for 25 (*B2M*) or 35 (*CYYR1 *CAG^- ^or CAG^+^) cycles (30 sec at 94°C, 30 sec at 63°C, 45 sec at 72°C), and a final extension for 7 min at 72°C.

In a first experiment, 32 NE tumor samples were studied along with the standard set of 8 normal tissues (see the paragraph *RNA sources *above). In a second experiment, 17 NE tumor samples (nn. NE 1, NE 2, NE 4–8, NE 10–12, NE 14, NE 18, NE 20, NE 25, NE 27, NE 29–30 in the Additional file 1) from the liver, pancreas and breast were compared with the above described second control set of 2 normal tissues and 2 non-neuroendocrine tumor samples. In this case, the PCR cycles for *CYYR1 *isoforms were 40.

### Sequence analysis

The *CYYR1 *CDS RT-PCR products obtained as described above were gel analyzed following standard methods [7], purified using GenElute PCR Clean-up kit (SIGMA, St. Louis, MO), and then subjected to automated sequence analysis of both DNA strands for each fragment, with the same primers used in the PCR reactions. The BigDye chain-terminator method was used with an automated ABI 310 DNA sequencer (Perkin-Elmer, Foster City, CA).

Point mutation in sample NE 16 was confirmed by four independent RT-PCR reactions.

### Enzymatic digestion

*CYYR1 *CAG^+ ^isoform sequence presents a PstI restriction site CTGCA|G, where the vertical bar indicates the cut position, so PstI was used to selectively digest the *CYYR1 *CAG^+ ^amplification product. Enzymatic digestions of *CYYR1 *CDS RT-PCR products from normal brain and one NE tumor (sample NE 16) obtained with primers #1 and #2 were performed in 20 μL final volume, containing 200–300 ng amplicon and 13 U PstI enzyme (SIGMA, St. Louis, MO) with 1× buffer H. An overnight incubation at 37°C was performed. The digested products (10 μL) were separated into 1.5% agarose TBE (Tris-Borate-EDTA) gel stained with ethidium bromide and detected under ultraviolet light.

### Gel imaging and quantitative relative analysis

For quantitative relative analysis, 10 μL aliquots of each PCR product were separated into 1.5% agarose TAE (Tris-Acetate-EDTA) gels. *B2M*, *CYYR1 *CAG^- ^and *CYYR1 *CAG^+ ^RT-PCR products from the same RNA samples were separated into the same gel. Marker M5 (Fermentas, Hanover, Maryland, MD) at two different dilutions was used as a quantitative reference. After separation, the gels were stained in TAE buffer containing ethidium bromide (0.5 μg/mL) and detected under ultraviolet light in "unsaturated" pixel modality with the Gel Doc 2000 Imaging System. Digital images were quantified and analyzed using Quantity One software (Bio-Rad, Hercules, CA, USA).

Intensity values of the PCR product bands were calculated in comparison with a regression line, with the correlation coefficient ≥ 0.99, generated from measurements of at least four Marker M5 bands of different concentration values. In particular, we used the "Volume Rect Tool" function to acquire pixel intensity data for each band. The gel image background was always subtracted.

### Statistical analysis

The mean for each replicate data point and, in order to normalize the *CYYR1 *expression level, the *CYYR1*/*B2M *product mass ratio were determined. The statistical analysis was performed using JMP software, ver. 5 (SAS Institute, Cary, NC, USA). The unpaired t-test was used to compare normalized *CYYR1 *expression levels between normal and tumoral tissues, as well as to compare relative expression of *CYYR1 *CAG^- ^with CAG^+ ^isoforms.

The ANOVA test was performed to compare *CYYR1 *expression levels (for CAG^- ^isoform, CAG^+ ^isoform and CAG^-^/CAG^+ ^ratio) among each different hystological subclass of the tumors studied in the first experiment.

Differences were considered significant with *p *< 0.05.

### Bioinformatic analysis

In order to study the evolution of "subtle" splicing [8] of the *CYYR1 *mRNA (CAG^- ^and CAG^+ ^isoforms), *CYYR1 *mRNA encompassing the point alternatively spliced – [GenBank:NM_052954] from base 556 to 757 – as well as the CYYR1 product amino acid sequence were analyzed by BLAST (Basic Local Alignment Search Tool) family programs with default parameters. This was done using the following GenBank divisions: "nr" (non redundant), "human ESTs", "mouse ESTs" and "other ESTs" database sequences. The same protocol was used to search for sequences harboring the new mutation described in one NE sample.

## Results

### RT-PCR – sequence analysis

The RT-PCR amplification products for *CYYR1 *CDS mRNAs were successfully obtained from all the 32 NE RNA samples. In all cases, gel electrophoresis analysis revealed a single band of the expected size.

Electrophoretograms showed a peak frameshift following the boundary between exon 3 and exon 4, consistently observed in both forward and reverse directions of the sequencing reaction. Visual analysis of the peaks suggested the simultaneous presence of two sequences differing by a three-base insertion in all of the samples analyzed (data not shown).

**Figure 1:**
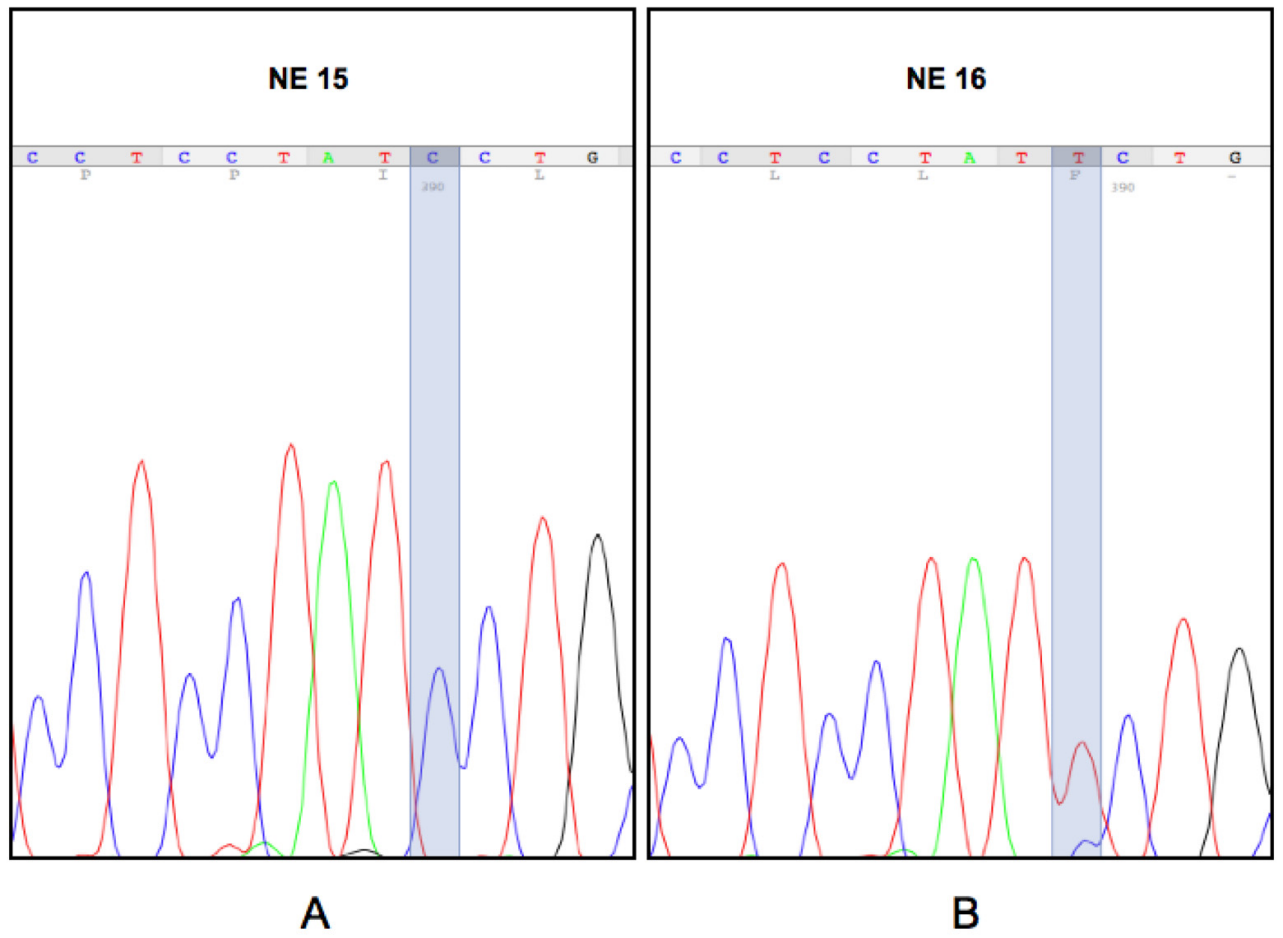
***CYYR1 *mutation in an NE tumor sample**. Representative electrophoretograms of a sample with normal sequence NE 15 (A) and the mutated sample NE 16 (B), where a variation (apparently in heterozygotic form) in position 333 of CDS (T replaces C) leads to a P111S amino acid change.

In addition, the *CYYR1 *CDS sequence did not show differences from the GenBank reference in any of the analyzed samples except in sample NE 16, where a variation (apparently in heterozygotic form) in position 333 of CDS (T replaces C) led to a P111S amino acid change (Figure 1).

A single nucleotide polymorphysm (SNP) at position 201 with respect to reference CDS (C replaces T at third position of codon 67, exon 3) was observed in heterozygosis (15 cases: NE 1, NE 4–9, NE 12, NE 15, NE 16, NE 18–20, NE 23, NE 31) or homozygosis (8 cases: NE 17, NE 24–27, NE 29, NE 30, NE 32). This base substitution transforms the codon 67 GTT into the codon GTC; in both cases, the coded amino acid is valine (V). This polymorphysm was present in the single nucleotide polymorphysm database (dbSNP) at NCBI (National Center for Biotechnology Information) [9] as cluster rs966410 (heterozygosity: 0.492).

### Enzymatic digestion

**Figure 2:**
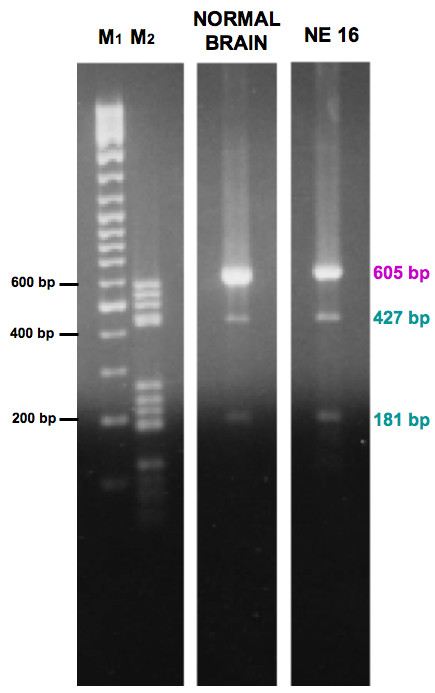
**Enzymatic digestion of *CYYR1 *CDS RT-PCR products from normal brain and an NE tumor**. Gel electrophoresis analysis of *CYYR1 *CDS RT-PCR products, from normal brain and NE 16, after PstI digestion. Expected size bands (CAG^- ^form: 605 bp, CAG^+ ^form: 427 bp and 181 bp) were obtained in both samples. M1: GeneRuler marker, 500 ng; M2: DNA M5 marker, 250 ng.

In order to confirm the presence of two isoforms, an enzymatic digestion of *CYYR1 *CDS RT-PCR products from normal brain and one NE tumor (sample NE 16) was performed with PstI enzyme, specific for *CYYR1 *CAG^+ ^isoform digestion. Expected size bands (CAG^- ^form: 605 bp, CAG^+ ^form: 427 bp and 181 bp) were obtained in both samples (Figure 2).

### Quantitative relative RT-PCR analysis

**Figure 3:**
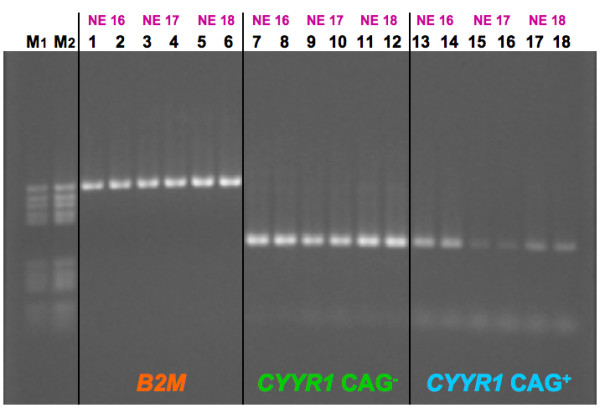
**RT-PCR of *CYYR1 *mRNA isoforms**. An example of agarose gel loaded with duplicated RT-PCR products: *B2M *(lanes 1–6, 586 bp), *CYYR1 *CAG^- ^(lanes 7–12, 321 bp), and *CYYR1 *CAG^+ ^(lanes 13–18, 324 bp) mRNAs for samples NE 16, NE 17 and NE 18, respectively from left to right. DNA M5 marker (M1 and M2, 250 ng and 500 ng, respectively) were used for quantification by Gel Doc 2000 software.

The RT-PCR amplification products for *B2M*, *CYYR1 *CAG^- ^and *CYYR1 *CAG^+ ^mRNAs were successfully obtained in duplicate from 29 NE RNA samples and from 8 normal RNA controls. 3 out of 32 cases (NE 13, NE 24 and NE 28) were not considered in the analysis due to failure in obtaining data in duplicate. In all cases, gel electrophoresis analysis revealed single bands of the expected size (Figure 3).

The gel images acquired in UV light and in "unsaturated pixel" mode were analyzed. RT-PCR products of *B2M*, *CYYR1 *CAG^- ^and *CYYR1 *CAG^+ ^mRNAs obtained from the same sample were electrophoresed in the same gel (Figure 3).

This process generated two replicate data points, expressed as PCR product ng, which were used for subsequent elaborations.

Duplicate products for each gene were compared to evaluate the method reliability. In the first experiment, the mean percentage of difference between the two replicate measurements and the respective mean value for 29 NE tumor samples and 8 normal tissues was: 3.6% (tumors) or 2.5% (normal samples) for *B2M*, 3.8% (tumors) or 3.1% (normal samples) for *CYYR1 *CAG^-^, and 15.8% (tumors) or 8.9% (normal samples) for *CYYR1 *CAG^+^. The percentage of difference between the two replicate measurements and the respective mean value was lower than 13% for all the genes in all the samples, except for the *CYYR1 *CAG^+ ^isoform values in 10 tumor samples and 1 normal sample, where the 16.3–61.2% range difference was due to the presence of values situated near to the lowest detectable level. The mean value of the two measurements was then routinely used in the statistical comparisons.

In the second experiment, the mean percentage of difference between the two replicate measurements and the respective mean value for 17 NE tumor samples and 4 control samples (2 non-neuroendocrine tumors and 2 normal tissues) was: 4.0% (NE tumors) or 3.8% (control samples) for *B2M*, 4.8% (NE tumors) or 5.8% (control samples) for *CYYR1 *CAG^-^, and 16.2% (13 NE tumors) or 13.1% (control samples) for *CYYR1 *CAG^+^. Inconsistent duplicate measurements for *CYYR1 *CAG^+ ^isoform were observed in 4 samples, due to values being in the lowest range of detection, so these samples (NE 10, NE 12, NE 25, and NE 30) were not considered for further analysis. The percentage of difference between the two replicate measurements and the respective mean value was lower than 15% for all the genes in all the samples, except for *B2M *in one NE tumor sample and the *CYYR1 *CAG^+ ^isoform values in 5 tumor samples and 1 control sample, where the 17.4–54.8% range difference was due to the presence of values situated near to the lowest detectable level. The mean value of the two measurements was then routinely used in the statistical comparisons. In 7 cases (6 NE tumors and 1 normal tissue sample), the CAG^+ ^isoform value was under the minimum detectable (0.25 ng) and it was considered 0.24 to allow calculations, with results analogous to those obtained when the corresponding samples were omitted from the statistical analysis.

### Statistical analysis

All differences among *CYYR1 *mRNA isoform expression levels refer to RT-PCR product mass, normalized as described in the "Methods" section.

In the first experiment, assessable replicate data points for *B2M*, *CYYR1 *CAG^- ^and CAG^+ ^mRNAs were successfully obtained for 29 out of 32 total samples tested; NE 13, NE 24 and NE 28 were not evaluated due to technical problems. The difference in the *CYYR1 *CAG^-^/CAG^+ ^mRNA isoform ratio between tumors and normal tissues was not statistically significant (mean ± standard deviation: tumors (n = 29), 19.3 ± 32.6; normal tissues (n = 8), 10.9 ± 6.3). The *CYYR1 *CAG^- ^expression level was significantly lower in tumor samples in comparison with normal tissues at an approximate ratio of 2:3 (*p *= 0.022; mean ± standard deviation: tumors, 0.92 ± 0.25; normal tissues, 1.31 ± 0.76), while the difference in the *CYYR1 *CAG^+ ^expression level between tumors and normal tissues was not significant (mean ± standard deviation: tumors, 0.15 ± 0.13; normal tissues, 0.15 ± 0.10). No statistical difference was observed in either CAG^- ^or CAG^+ ^isoform expression, or between macrodissected (n = 11) and non-macrodissected (n = 18) NE sample subgroups (see Additional file 1).

In the second experiment, the difference in the *CYYR1 *CAG^-^/CAG^+ ^mRNA isoform ratio between NE tumors (n = 13) and control tissues (2 non-neuroendocrine and 2 normal corresponding tissues; n = 4) was not statistically significant (mean ± standard deviation: NE tumors (n = 13), 321.0 ± 359.0; control tissues (n = 4), 80.8 ± 138.7). The difference in the *CYYR1 *CAG^- ^expression level (mean ± standard deviation: NE tumors (n = 17), 0.43 ± 0.17; control tissues (n = 4), 0.57 ± 0.18) as well as the difference in the *CYYR1 *CAG^+ ^expression level (mean ± standard deviation: NE tumors (n = 13), 0.016 ± 0.021; control tissues (n = 4), 0.05 ± 0.06) between NE tumors and control tissues was not significant. No statistical difference was observed in either CAG^- ^or CAG^+ ^isoform expression, or between macrodissected (CAG^-^, n = 8; CAG^+^, n = 6) and non-macrodissected (CAG^-^, n = 9; CAG^+^, n = 7) NE sample subgroups (see Additional file 1).

Differences among subclasses defined according to the WHO classification were investigated in the 29 samples for which data were obtained in the first experiment (15 WDEC, 11 PDEC and 3 MEET samples) using the ANOVA test, and they were not statistically significant for CAG^-^, CAG^+ ^and CAG^-^/CAG^+ ^*CYYR1 *isoform expression levels.

In all samples studied, the *CYYR1 *CAG^- ^isoform was expressed at an higher level than CAG^+ ^isoform. The difference was highly statistically significant when comparing the respective expression levels for each isoform in the first experiment (29 NE tumor samples, *p *< 0.01; 8 normal tissues, *p *< 0.01) as well as in the second experiment (13 NE tumor samples, *p *< 0.01; 4 control tissues, *p *< 0.01).

### Bioinformatic analysis

Bioinformatic analysis was conducted using database versions available in April 2006. In "nr" database, 6 human mRNA sequences encompassing the variant splice point were found: 5 of which related to the CAG^- ^first described isoform. In "human ESTs" database, 14 mRNA sequences relating to *CYYR1*, assignable to one of the two isoforms, were identified: 6 entries with CAG^+ ^sequence and 8 entries with CAG^- ^sequence (see Additional file 2).

**Figure 4:**
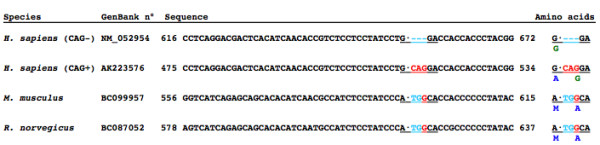
***CYYR1 *exon 3/exon 4 junction and relative amino acids codified**. Alignment of human *CYYR1 *mRNA sequence, encompassing the CAG^-^/CAG^+ ^with the corresponding mRNA sequences from other species. The symbol • is used to indicate the exon/exon junction. Predicted amino acid is shown aligned to the first base of its codon; **G **= glycin, polar not charged; **A **= alanine and **M **= methionine, not polar.

Figure 4 shows the alignment of the *CYYR1 *gene family nucleotide sequences present in the GenBank database and encompassing the CAG^-^/CAG^+ ^exon junction. We considered only species for which at least one mRNA sequence or two EST sequences were available, and this homology was significant at least in allowing alignment at nucleotide level using BLAST. Exon 4 of *Mus musculus *[GenBank:BC099957] and *Rattus norvegicus *[GenBank:BC087052] *CYYR1 *begins with the TGG sequence, which does not offer a second splicing signal, unlike the CAG sequence at the beginning of human *CYYR1 *CAG^+ ^exon 4 [GenBank:AK223576].

**Figure 5:**
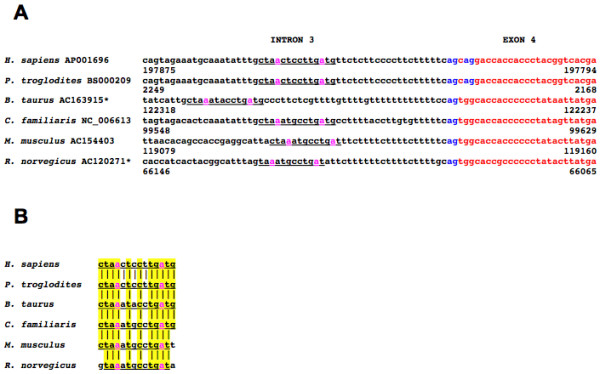
***CYYR1 *intron 3/exon 4 boundary**. Comparison of *CYYR1 *57 bases at 3' of intron 3 and 25 bases at 5' of exon 4 in some mammalian species. The symbol * indicates GenBank sequences from ''htgs'' (draft) division. In blue: splicing sites; in magenta: putative branch points (A). In B: comparison of the putative branch sites (the underscored regions in A); in yellow: high identity regions.

The genomic sequences available for this junction all come from mammalian species, and we compared 57 bases at 3' of intron 3 and 25 bases at 5' of exon 4 (Figure 5A). The sequences of *Bos taurus *[GenBank htgs:AC163915], *Canis familiaris *[GenBank:NC_006613], *Mus musculus *[GenBank:AC154403] and *Rattus norvegicus *[GenBank htgs:AC120271] *CYYR1 *at genomic level are clearly not consistent with the possibility of an alternative splicing as is possible in humans, because only one CAG sequence, therefore one acceptor splice site (AG), is present at the 3' intronic boundary, and the next exon begins with the TGG sequence (CAG//TGG, where//indicates intron/exon boundary).

In the anthropomorphic monkey *Pan troglodytes *(Chimpanzee) [GenBank:BS000209] as well as in *Homo sapiens *[GenBank:AP001696] the genomic sequence corresponding to intron 3/exon 4 boundary is CAGCAG. In this case, at the intron/exon junction, there are two successive splice sites separated by three bases (CAG//CAG) (Figure 5A).

This genomic region is highly conserved, and it contains a similar putative branch site upstream of polypyrimidine tracts of various length and composition (Fig. 5B).

**Figure 6:**
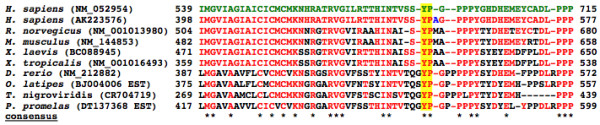
**CYYR1 protein sequences**. Manual alignment of the vertebrate CYYR1 protein sequences available in GenBank (nr or EST divisions). Only the partial amino acid sequence encompassing the sequence corresponding to the CAG^+^/CAG^- ^exon junction is shown. The highly conserved YP amino acids are highlighted in yellow. In blue the alanine (A) in the *CYYR1 *CAG^+ ^isoform.

Figure 6 shows the alignment of the vertebrate CYYR1 putative protein sequences available in GenBank (nr or EST divisions) or inferred by nucleotide sequences available (see Additional file 2). Alignment underlines that the encoded amino acids corresponding to the CAG^-^/CAG^+ ^exon junction in the *CYYR1 *mRNA are well conserved from amphibians to bovines. Two amino acids conserved in all analyzed species (YP) are always followed by a couple of amino acids, one of which is always alanine (A): the same amino acid encoded by the rarer human mRNA isoform CAG^+^. In fish, a conserved block is found downstream of the CAG^-^/CAG^+ ^exon junction.

No sequence with NE 16 mutation was found in the analyzed databases.

## Discussion

Neuroendocrine cells in the organism are part of a diffuse system of cells with neuroendocrine features characterized by ultrastructural features (presence of dense core granules of 100–400 nm diameter) and immunohistochemical phenotypes (pan-neuroendocrine markers and specific hormonal products) [10]. We first described *CYYR1 *gene as a novel locus on human chromosome 21, encoding a product with no similarity to any known protein. Based on the observation that the *CYYR1 *gene appears to be expressed, as evaluated by EST database analysis, in several types of neuroendocrine cells, we decided to investigate *CYYR1 *sequence and expression in a large series of human NE tumors. We also wanted to obtain new data about the *CYYR1 *gene, which has never been studied in-depth and whose product function is, at the moment, unknown.

Sequence analysis of the 32 NE samples tested showed a CDS sequence identical to that previously described in normal subjects, except for a case in which we identified a point mutation. The mutation is a C-T transition, apparently in heterozygotic state, leading to a P111S change predicted in the encoded product. This change is not present in any human sequence obtained from either finished sequence or EST databases. The replaced proline amino acid is situated one position before the first PPPY motif, which is present in three copies in the carboxy-terminus of the CYYR1 protein. This motif is known to be present in some viral proteins, where it is required for virus budding [11], and it is also described in proteins involved in interactions between cytoskeleton and extracellular matrix [12,13]. Due to the general relevance of proline for protein structure, and to its proximity to a functional motif, it may be speculated that this change could affect the CYYR1 function. Interestingly, the only NE mutated sample among those analyzed is a tumor arising in the neck region, a less common site of origin of neuroendocrine tumors.

In addition, a known SNP has been found in some samples: a C-T change at the third position of codon 67.

Sequence analysis was also useful to show an unexpected "subtle" splicing isoform of *CYYR1 *mRNA, derived from the alternative use of an AG acceptor splice site located at the 3' end of *CYYR1 *intron 3, leading to the generation of mRNA isoforms which differentiate by only three bases. This new isoform encodes a predicted product with an adjunctive alanine (A) amino acid at position 112, located just before the glycine (G) preceding the first PPPY motif (Figure 6). Due to its position, it is likely that this adjunctive amino acid could affect CYYR1 function, allowing the presence of two protein products whose relative expression is regulated by alternative splicing. Alternative splicing leading to mRNA isoforms encoding slightly different polypeptides had been reported only anecdotally until a recent systematic survey [8]. This work has demonstrated the relative frequency of this phenomenon in several vertebrate genomes, underlining its biological relevance as a means to increase functional complexity generated by the same locus sequence.

Systematic analysis by RT-PCR proved that both isoforms are expressed in all 29 NE and 8 normal tissue samples investigated, with the constant prevalence of the CAG^- ^isoform, as also confirmed by human EST database analysis. We also accurately quantify the relative expression of the two forms, using an approach whose reliability we have previously described [14]. We found that the most commonly expressed isoform is the CAG^- ^isoform, the first described mRNA for *CYYR1 *locus [1]. The ratio between the expression of the two isoforms shows a greater variability with respect to a comparable gene such as the insulin-like growth factor 1 receptor (*IGF1R*). We previously found that *IGF1R *undergoes a "subtle" splicing with a tight regulation in both normal and neoplastic cells, leading to a constant 3:1 ratio [14]. However, when organs similar to those in which tumors arose were used as controls, statistical analysis failed to identify a significant difference in the *CYYR1 *CAG^-^/CAG^+ ^ratio, as well as in the normalized CAG^+ ^or CAG^- ^mRNA level, between NE and normal samples. The EST database seems to support, for several tissues, a high expression of *CYYR1 *in some neuroendocrine tumors. For example, among the 19 *CYYR1*-related ESTs with "lung" origin, 12 are derived from lung carcinoid, and among the 12 ESTs with "pancreas" origin, 11 are derived from insulinoma. However, 10 of the lung carcinoid ESTs derive from the same library, as well as all the insulinoma ESTs. In general, EST database may provide indications about gene expression profile, but data are of limited statistical value.

To investigate the biological relevance of the "subtle" splicing of *CYYR1 *mRNA, we performed a bioinformatic analysis of all available sequence databases. *CYYR1 *locus "subtle" splicing has actually been observed only in *Homo sapiens*, while in *Pan troglodytes *the genomic sequence is consistent with its existence.

## Conclusion

We have described a new "subtle" splicing isoform for *CYYR1 *locus and alterations of sequence and expression of this gene in a large series of NE tumors. The *CYYR1 *mRNA isoform expression level was comparable in tumor samples and normal tissues, and a missense mutation was identified in one tumor sample. The *CYYR1 *CAG^- ^isoform was significantly more expressed than the CAG^+ ^isoform in NE tumor as well as in control samples investigated. Further investigations are necessary to clarify the functional role of CYYR1 products in both normal and neoplastic cells.

## Abbreviations

B2M, beta-2 microglobulin; BLAST, basic local alignment search tool; BLASTN, Blast nucleotide-nucleotide; cDNA, DNA complementary to RNA; CDS, coding sequence; dbSNP, single nucleotide polymorphism database; dNTP, deoxyribonucleotide triphosphate; DNES, diffuse neuroendocrine system; EST, expressed sequence tag; htgs, high throughput genomic sequences; IGF1R, insulin-like growth factor 1 receptor; mRNA, messenger RNA; NCBI, National Center for Biotechnology Information; NE, neuroendocrine; nr, non redundant; RT-PCR, reverse transcription – polymerase chain reaction; SNP, single nucleotide polymorphism; TAE, tris-acetate-EDTA; TBE, tris-borate-EDTA.

## Competing interests

The author(s) declare that they have no competing interests.

## Authors' contributions

LV designed the study, carried out the molecular genetic studies, participated in the sequence alignment and drafted the manuscript. FFr discussed the molecular genetics and bioinformatics data. SAH prepared and provided the NE tumor biological samples. SC and RC participated in molecular genetics analysis and result interpretation. LL and FFa were involved in statistical and bioinformatics data generation and interpretation. PC and MZ partecipated in study coordination and supervision. DC prepared and provided the NE tumor biological samples and participated in data discussion and drafting the whole manuscript. PS performed the statistical analysis and participated in study design and discussion. All authors read, discussed and approved the final manuscript.

## Pre-publication history

The pre-publication history for this paper can be accessed here:



## Supplementary Material

Additional file 1 **RNA sample list**. Patients and samples summary data table. Click here for file

Additional file 2 **GenBank accession numbers of *CYYR1 *mRNA-related sequences found by bioinformatic analysis**. Table of all non redundant or EST sequence accession numbers of *CYYR1 *mRNA related sequences available in NCBI databases in April, 2006. Click here for file
