# Supplementary material for: Sequence, "subtle" alternative splicing and expression of the CYYR1 (cysteine/tyrosine-rich 1) mRNA in human neuroendocrine tumors
Source: BMC Cancer. 2007 Apr 18;7:66. doi: 10.1186/1471-2407-7-66 (PMC1863428; doi:10.1186/1471-2407-7-66)
Supplement: Additional file 1 — RNA sample list. Patients and samples summary data table. [file 1471-2407-7-66-S1.doc]

**RNA sample list.**

WDEC, well-differentiated endocrine carcinoma; PDEC, poorly differentiated endocrine carcinoma; MEET, mixed exocrine-endocrine tumor. Rt: right; Lt: left; HN: head and neck. In the case of macrodissected tissues, the percentage of tumor (not stromal) cells is given in parenthesis. Sample groups NE 2-3, NE 4-6 and NE 7-9 were obtained from three different patients, respectively.

| **Sample** | **Age/Sex** | **Classification** | **Site** |
| --- | --- | --- | --- |
| NE 1 | 75 / M | WDEC (metastatic) | Liver, Rt |
| NE 2 | 59 / F | WDEC (metastatic) | Liver, Lt lobe |
| NE 3 | 59 / F | WDEC (metastatic) | Bowel, small |
| NE 4 | 64 / F | WDEC (metastatic) (100%) | Liver, Rt |
| NE 5 | 64 / F | WDEC (metastatic) (98%) | Liver, Lt |
| NE 6 | 64 / F | WDEC (metastatic) (98%) | Liver, Rt |
| NE 7 | 66 / F | WDEC (metastatic) (98%) | Liver |
| NE 8 | 66 / F | WDEC (metastatic) (100%) | Liver |
| NE 9 | 66 / F | WDEC (metastatic) (95%) | Colon, involving muscularis propria |
| NE 10 | 70 / M | WDEC (95%) | Pancreas |
| NE 11 | 60 / F | PDEC (large cell neuroendocrine carcinoma) | Breast, Rt |
| NE 12 | 64 / M | MEET (metastatic) (85%) | Liver, segment 7 and 8 |
| NE 13 | 60 / M | PDEC (100%) | Omental nodule |
| NE 14 | 55 / M | PDEC (metastatic) (100%) | Liver |
| NE 15 | 79 / M | PDEC | Abdominal perianal mass |
| NE 16 | 61 / F | MEET | Neck, Rt (HN) |
| NE 17 | 59 / M | PDEC (small cell neuroendocrine carcinoma) | Parotid, Rt, temporal (HN) |
| NE 18 | 63 / F | PDEC | Breast, Rt |
| NE 19 | 79 / M | PDEC | Groin |
| NE 20 | 69 / M | PDEC (metastatic) | Liver, Lt lobe |
| NE 21 | 70 / F | PDEC | Ovary, Rt |
| NE 22 | 60 / F | PDEC (metastasis from breast) | Peristernal mass |
| NE 23 | 64 / M | MEET | Lung, Lt |
| NE 24 | 66 / F | PDEC | Breast, Rt |
| NE 25 | 54 / M | WDEC | Pancreas |
| NE 26 | 56 / M | PDEC (metastatic, consistent with pancreatic primary tumor) | Liver |
| NE 27 | 48 / M | WDEC | Pancreas (head) |
| NE 28 | 77 / F | MEET | Pancreas |
| NE 29 | 52 / M | WDEC (gastrinoma) | Pancreas |
| NE 30 | 70 / M | WDEC | Pancreas |
| NE 31 | 75 / M | WDEC (100%) | Pancreas |
| NE 32 | 43 / F | PDEC (metastasis from islet cell tumor) (100%) | Abdominal wall |
