# Supplementary material for: Sequence, "subtle" alternative splicing and expression of the CYYR1 (cysteine/tyrosine-rich 1) mRNA in human neuroendocrine tumors
Source: BMC Cancer. 2007 Apr 18;7:66. doi: 10.1186/1471-2407-7-66 (PMC1863428; doi:10.1186/1471-2407-7-66)
Supplement: Additional file 2 — GenBank accession numbers of CYYR1 mRNA-related sequences found by bioinformatic analysis. Table of all non redundant or EST sequence accession numbers of CYYR1 mRNA related sequences available in NCBI databases in April, 2006. [file 1471-2407-7-66-S2.doc]

**GenBank accession numbers of *CYYR1* mRNA-related sequences found by bioinformatic analysis.**

Source tissue is given in parenthesis when known. NM_ sequences are given in round brackets under species; species with XM_ sequences or only one EST sequence are not referred.

| **Database:** | **non redundant** | | **ESTs** | |
| --- | --- | --- | --- | --- |
| **Isoform:** | CAG- | CAG+ | CAG- | CAG+ |
| *Homo sapiens*  (NM_052954) | AL833200 (lymph node);  AF401639 (brain);  AY061853 (?);  AK054581 (neuroblastoma);  BC036761 (ovary) | AK223576 (heart) | DB268533 (uterus);  BP197057 (adrenal gland);  CV030874 (mixed);  BX360059 (placenta);  BM546023 (ovary);  BF243436 (acute myelogenous leukemia);  BQ329249 (lung);  BQ889207 (dorsal root ganglia) | BU853259 (testis);  DA747989 (teratocarcinoma);  DN992654 (brain);  BF676689 (prostate);  DB265471 (uterus);  BI762766 (pooled colon, kidney, stomach) |
| **Database:** | **non redundant** | | **ESTs** | |
| *Mus musculus*  (NM_144853) | BC099957 (pancreas);  AF442733 (brain);  AY061854 (?);  AK031840 (medulla oblongata) | | CF584057 (pancreas);  AV327062 (medulla oblongata) | |
| *Rattus norvegicus*  (NM_001013980) | BC087052 (lung) | | CK475557 (lung);  AW917652 (mix - brain, ovary, placenta, kidney, lung, liver, embryo, heart, muscle, spleen) | |
| *Xenopus laevis* | BC088945 (egg) | | DV034197 (cornea);  BP714774, BP699289, BP693926, BP693629, BP691092, BP677669, BP732233 (anterior neuroectoderm, late gastrula);  CO383025 (dorsal blastopore lip);  BX849711 (?);  BJ638286, BJ622637, BJ621561, BJ638542 (whole embryo, early gastrula);  CA973412, CA972992 (egg);  BE026256 (unfertilized egg);  BU899894 (ovary) | |
| *Xenopus tropicalis*  (NM_001016493) | CR760825 (tadpoles) | | CX460433, CX479824, CX479823, CX460432, CX470959, CX470958 (whole embryo - gastrula);  CX968780, CX968779 (intestine - adult);  CX414894, CX414893 (whole embryo - tadpole);  CR567312 (tailbud-head);  CR426841, CR444244 (tailbud);  CN107081, CN106237 (pool of heads and retinas from tailbud);  BX772429 (egg);  AL593882, BX750329 (gastrula);  BX729530, BX728190, BX728189, BX710302, BX710301 (tadpole);  AL655906, BX692027, AL788783 (neurula) | |
| *Danio rerio*  (NM_212882) | AY690837 (embryo);  BC066606 (embryo, 24 hours post fertilization) | | CT700616, CT644209 (myoblast);  CD754013 (embryo) | |
| *Oryzias latipes* | - | | BJ004006, BJ007269 (whole embryo);  AM150126 (gastrula) | |
| *Tetraodon nigroviridis* | CR704719 (eyes) | | - | |
| *Pimephales promelas* | - | | DT137367, DT137368 (whole adult) | |
